# Factors associated with contracting border malaria: *A systematic and meta-analysis*

**DOI:** 10.1371/journal.pone.0310063

**Published:** 2025-01-03

**Authors:** Tichaona Fambirai, Moses Chimbari, Tafadzwa Mhindu

**Affiliations:** School of Nursing and Public Health, College of Health Sciences, Howard College Campus, University of KwaZulu-Natal, Durban, South Africa; Clinton Health Access Initiative, UNITED STATES OF AMERICA

## Abstract

Vector resistance, human population movement, and cross-border malaria continue to pose a threat to the attainment of malaria elimination goals. Border malaria is prominent in border regions characterised by poor access to health services, remoteness, and vector abundance. Human socio-economic behaviour, vectoral behaviour, access and use of protective methods, age, sex, and occupation have been identified in non-border regions as key predictors for malaria. We conducted a systematic and meta-analysis review to characterise and establish pooled effect sizes of the factors associated with the occurrence of border malaria. An exhaustive search was done in EBSCOHost (Medline Full Text), Health Source, Google Scholar, Regional Office for Africa Library, African Index Medicus, and PubMed databases. A total of 847 articles were identified from the search and after screening for quality and eligibility, twelve (12) articles were included in the review. Pooled odds ratios, inverse variance statistic (I^2)^, Luis Furuya-Kanamori (LFK) index, and forest plot were computed. Findings from this study suggest night outdoor activities (POR 2.87 95% CI, 1.17 7,01), engaging in forestry activities (POR 2.76 95% CI, 2.08 3.67), working in mines (POR 197 95% CI, 175 22171), access to poor housing structure (POR 3.42 95% CI, 2.14 5.46), and cross-border movement (POR 50.86 95% CI, 12.88 200.85) none use of insecticide-treated nets (POR 5.09 95% CI, 2.44 10.63) were all significantly associated with contracting malaria within border regions. The use of insecticide-treated nets (ITN) (POR 0.61 95% CI, 0.50 0.76) and indoor residual spraying (IRS) (POR 0.61 95% CI, 0.47 0.79) were protective. Risk factors for border malaria are comparable to non-border malaria. Effective border malaria control requires an integrated and targeted approach that addresses socio-economic, environmental, and behavioural drivers. Established vector control interventions remain protective and should be sustained to mitigate the border malaria burden effectively. Novel strategies should be developed to address the unique challenge of cross-border human population movement underpinned by robust regional, bilateral, and multi-sectoral collaborative initiatives.

## Introduction

Malaria continues to pose a global public health threat with over 240 million infections. Deaths attributable to malaria remain high with over 590 000 deaths recorded annually [1]. The majority of malaria cases and deaths (93%) are recorded in Sub-Sahara Africa and Asia [2, 3]. Since 2000, various global and regional interventions have contributed immensely to the significant reduction in the global malaria burden [3]. This decline may be attributed to intensified investment in vector control interventions, new anti-malaria medicines, and diagnostic methods [4]. Despite the gains in malaria control over the past two decades, border malaria poses a global threat to malaria burden reduction and elimination efforts [5, 6].

Malaria that occurs within, across, or along border regions, also termed “border malaria” or “cross border malaria”, remains a significant public health concern within border regions and countries nearing malaria elimination [5, 6]. Various factors have been implicated in the sustenance of border malaria, chief among them: limited access to health services, human mobility, and humanitarian crisis events [7]. Border regions are often characterised by a high population movement which facilitates parasite movement as malaria naïve population groups move from non-endemic areas to endemic regions predisposing them to risk of contracting malaria [8]. Human population movement (HPM) also links sub-regions with varying endemicity which can lead to sustained persistent malaria transmission. Evidence from Brazil’s border regions has shown that immigrants constitute the largest proportion of imported malaria cases [9]. Political instability leading to large-scale migration of displaced vulnerable populations has further contributed to the resurgence of malaria within the Ecuador–Peru border regions [10]. Illegal immigration for employment opportunities within South American regions has also been cited as the largest contributor to imported malaria cases [11]. In South East Asia (SEA) within the Mekong Delta sub-region; high transmission zones are associated with border regions [12]. Documented evidence shows the inhibiting factor of border malaria to achieving malaria elimination within SEA [13, 14]. Similar to the SEA scenario; high transmission zones within the Southern Africa region occur within the border regions of Mozambique, South Africa, Swaziland (Eswatini) [15, 16], and Zimbabwe [17]. Furthermore, surveillance data in South Africa has shown the significant contribution of imported and cross-border malaria to the local malaria burden along the Mozambique and Eswatini border regions [18, 19].

The abundance of malaria vectors within border regions has also been shown to be a key driver for malaria infections [20]. Malaria occurrence within border regions is further exacerbated by low socio-economic status, low developmental status, and political instability [21]. Whilst international border regions with endemic transmission pose complex scenarios in malaria control efforts [22], there are renewed global efforts to eliminate malaria guided by the Global Technical Strategy (GTS): 2016–2030. The overall goal of the GTS is to reduce malaria incidence by 90% and eliminate malaria in at least 35 countries by 2030 [4].

Substantial evidence exists on general risk factors for contracting malaria in non-border regions. However, risk factors for contracting border malaria have not been systematically characterised and evaluated for effect size and quality of evidence. Establishing the association between malaria and human behaviour and occupation in border areas is important to facilitate the formulation of targeted interventions. In this regard, we conducted a systematic review and meta-analysis aimed at aggregating available evidence on risk factors for contracting border malaria. Evidence from this study contributes to knowledge on border malaria that can be used to develop effective intervention strategies.

## Methods

**Fig 1:**
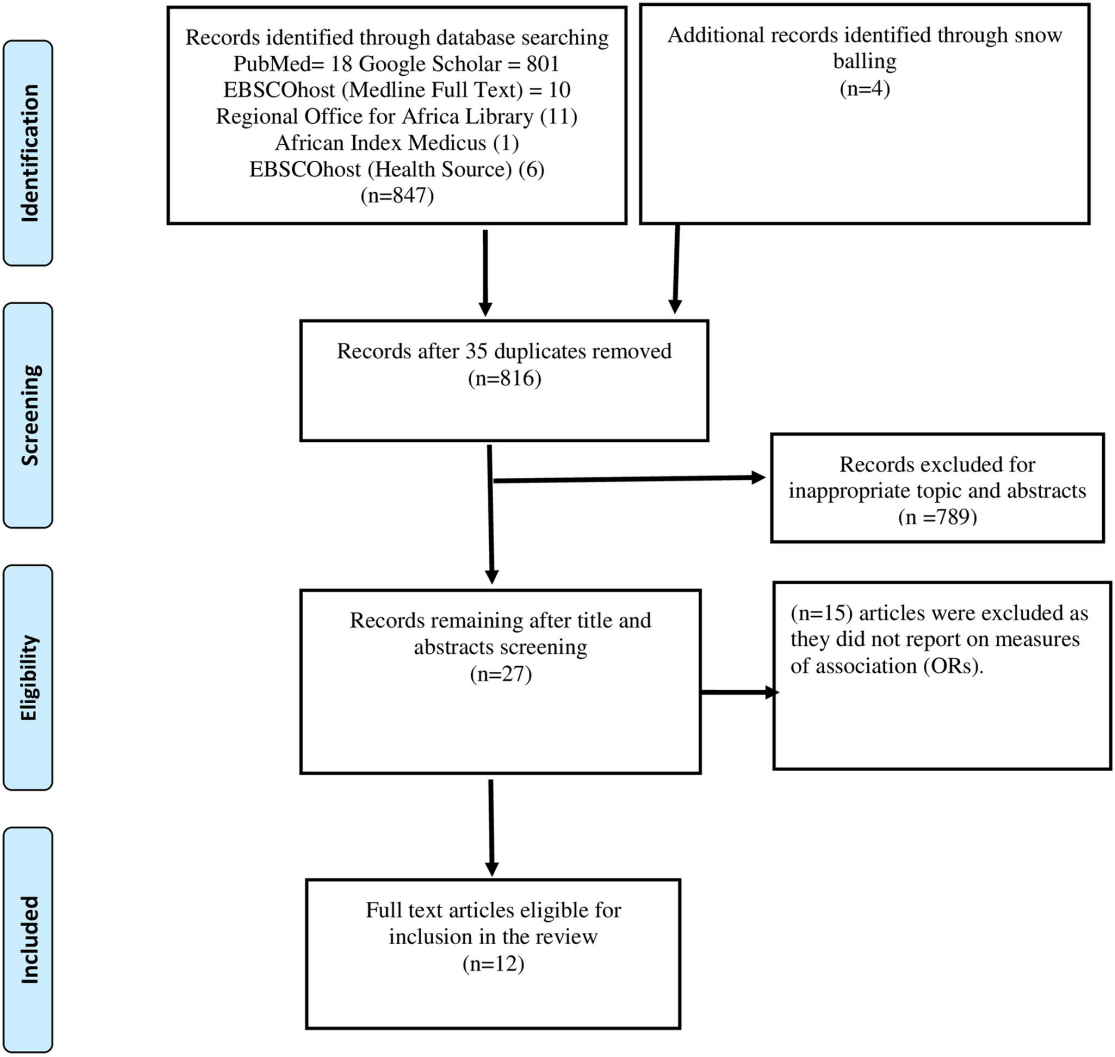
PRISMA diagram for studies and database searches.

We conducted a systematic literature search in PubMed, Google Scholar, EBSCOHost (Full Text Medline), EBSCOHost (Health Source), Regional Office for Africa Library (ROAL), and, African Index Medicus (AMI). The search considered studies in peer-reviewed journals published in English and focused on risk factors associated with cross-border malaria. The search used combinations of terms and Boolean operators: “Risk Factors” AND Malaria OR “Border Malaria” OR “Cross Border Malaria”. The last literature search for inclusion in this review was conducted on 26 April 2024. The study used the Preferred Reporting Items for Systematic Reviews and Meta-Analysis (PRISMA) guidelines [23] to search and select studies for inclusion in the review Fig 1. At the initial stage of identifying articles, titles, and abstracts were used to screen relevant papers. Relevant abstracts were further assessed for inclusion in the final articles list. Full articles were also assessed for results reporting Odds Ratios (OR) as a measure of associations.

### Quality of studies

We used the analytical cross sectional studies critical appraisal tool developed by Joanna Briggs Institute to assess the quality of the studies included in the review [24]. The tool uses a 8 -questionnaire assessment tool that assesses various attributes of a paper as shown in S1 Table.

The tool uses a score of 1 (for the presence of the attribute) and 0 for the absence of the attribute. The maximum score a paper could attain was 8. The final score was divided by 8 to get the quality index of each paper. Papers scoring (0.0–0.4) were classified as low quality, (0.5–0.7) moderate, and (0.8–1.0) high quality. Papers with a rating above 0.5 were included in the final analysis. TF and TM carried out data extraction. MJC handled the disagreements in the article’s critical appraisal rating.

### Inclusion and exclusion criteria

Articles published in English peer-reviewed journals, having been conducted in border regions and focused on factors associated with contracting malaria were included in the study. The inclusion criteria were: articles reporting data on measures of association OR/RRs with confidence intervals; and articles reporting on studies conducted in country border districts. Articles which did not report ORs were excluded from the study.

### Data extraction and synthesis

The following information was extracted from selected studies onto a Microsoft Excel template with the following headings: author and year of publication, decision and rationale for inclusion and exclusion S2 Table. The last data extraction was conducted in April 2024. TF and TM were responsible for the data extraction whilst MJC acted as a reviewer of the process and settled disagreements arising from the process. A further table was created to document all the exposure variables associated with border malaria from selected articles. The table captured author, year of publication, exposure of interest, odds ratio, and lower and upper 95% confidence intervals S3 Table. Articles reporting at least a single exposure variable and an outcome of interest (border malaria) together with odds ratios were captured on the datasheet. Articles with missing malaria exposure data and odds ratios were excluded from analysis.

### Data analysis

Data was synthesized quantitatively and qualitatively and presented in both narrative and tabular form to bring out the main findings on risk factors related to border malaria. Descriptive data as well as odds ratio extrapolated from the different papers were presented in tabular format and also explained in narrative form. Quantitative data was analysed using Meta XL (www.epigear.com) within Microsoft Excel (Microsoft^®^) to obtain pooled odds ratios (OR) and confidence intervals (CI) from selected studies. Meta XL uses a robust Inverse Variance Heterogeneity (IVhet) model because of its advantage of giving a correct probability at lower detected variance regardless of heterogeneity compared to the Random Effects Model and Fixed Effects Model [37]. IVhet allows computation of DOI plots to detect publication bias and also maintains correct coverage of probability at lower detected variance. In this meta-analysis, the outcome of interest was malaria infection across borders or within or along border regions. The independent variables were: age, sex, occupation, use of preventative measures, proximity to water bodies, housing structure, outdoor activity, and cross-border movement. To explore heterogeneity; sub-group analysis was also conducted through stratification of data according to gender, occupation, access to protective measures, housing structure, environment, outdoor behaviour, and cross-border movement. We also conducted a sensitivity analysis based on excluding non-significant risk factors from the subgroup analysis. The level of heterogeneity was evaluated using Cochrane’s Q statistics and inverse variance statistics (*I*^2^) [38]. Publication bias was assessed using the Luis Furuya-Kanamori (LFK) index on the Doi plot [37]. The I^2^ index was interpreted as no heterogeneity, low, moderate, and high if the values were 0, <25%, 50%, or >75%. An LFK index of +1 was deemed a symmetrical level of bias (no publication bias). An LFK index of +2 was classified as minor asymmetry (low publication bias). An LFK of greater than +3 was classified as major asymmetry (high publication bias). The forest plot was used to display estimated Odds Ratios and their 95% Confidence Intervals (CI).

## Results

Twelve (12) articles were selected for inclusion in this systematic and meta-analysis as shown in Fig 1. The studies that met the inclusion criteria were published between 2004 and 2022 in different regions as summarised in Table 1 and S1 Table. The studies were conducted in Eswatini [35], the Zimbabwe-Mozambique border region [25, 28] the Namibia-Angola border region [32, 36], Brazil-Venezuela border region [33], the China-Myanmar border region and the Thailand-Myanmar border region [26].

**Table 1:** Articles included in the systematic and meta-analysis.

Author & Year of Publication	Country/Region	Study Type
Kanyangarara et al. (2016) [25]	Zimbabwe-Mozambique region	case control
Tipmore et al. (2009) [26]	Thailand-Myanmar	cross-sectional
Zhao et al. (2018) [27]	China	cross-sectional
Kureya et al. (2017) [28]	Zimbabwe-Mozambique border	cross-sectional
Chavepojnkamjon et al. (2004) [29]	Thailand -Myanmar border region	case control
Xu et al. (2015) [30]	China (Yunnan Vietnam, Laos, and Cambodia border)	case-control
Li et al. (2013) [31]	China	retrospective case-control
Smith et al. (2021) [32]	Namibia-Angola border region	cross-sectional
Arisco J et al. (2021) [33]	Brazil (Venezuela and Guyana border region)	cross-sectional
Wangdi et al (2022) [34]	Brazil-Venezuela Border region	cross-sectional
Tejedor-Garavito et al. (2017) [35]	Swaziland (Eswatini)	cross-sectional
Smith et al. (2017) [36]	Namibia-Angola border region	case-control

### Gender and malaria

Table 2 shows that females were at greater risk of contracting malaria along the China-Myanmar border (OR 1.5 95%, CI 1.16–1.97) [27] compared to males. Along the Zimbabwe-Mozambique border [25], females were at a lower risk of contracting malaria compared to males (OR 0.9 95%, CI 0.82–0.97). In the Thai-Myanmar border region, males were significantly at risk of contracting malaria compared to females (OR 1,73 95%, CI 1.37–2.20) [26] as well as in Eswatini (OR 4.67 95% CI, 3.24–6.80) [35].

**Table 2:** Socio-demographic factors associated with border malaria.

Risk Factor	Positive association	Negative association	Non-significant association
Sex			
Female	1.51(1.16–1.97) [27]	0.9(0.82–0.97) (35)	0.91(0.53–1.54) [25]
Male	1.73(1.37–2.20) [26]		0.85(0.47–1.55) [26]
Age (years)			
<5	2.87(1.03–7.84) [25]		
5–14			2.64(0.53–5.-14) [25]
15–24			2.54(0.93–6.96) [25]
25–49			0.91(0.31–2.69) [29]
15–30			1.02(0.48–2.15) [26]
31–45	2.3(1–5.28) [26]		
5–15		0.57(0.46–7.1) [33]	
16–24	2.28(1.89–2.75) [26]		
25–40	2.63(2.2–3.16) [26]		
41–64	1.78(1.47–2.15) [26]		
65+		0.68(0.46–0.98) [26]	
Occupation			
Working in forest	3.28(1.96–14.79)		1.11(0.48–2.5) [29]
	3.55(1.20–10.33) [26]		
	2.66(1.95–3.62) [33]		
Mining	55.76(50.59–1.46) [33]		
Agriculture			0.22(0.82.-0.97) [33]
Agriculture activity at night	2.09(1.12–3.87) [32]		
Tourism	4.22(2.50–6.04) [33]		

### Age and malaria

Kanyangarara et al [25] and Tejedor-Garavito et al. [35] reported significant risk for contracting malaria for those aged under 5 years (OR 2,87 95% CI 1.03–7.87), (OR 24.74 95% CI 6.06–117.47). In the Eswatini study, Tejedor-Garavito et al. [35] reported a significant risk of contracting malaria for the following age ranges: 5–14 years (OR 8.9 95%, CI 2.15–42.79), 15–24 years (OR 5.79 95% CI 1.5–26.11), 25-44years (OR 8.21 95% CI, 2.22–35.61), 45-64years (OR 5.79 95% CI, 1.77–26.43). In Namibia-Angola border region, Smith et al. [36] also found significant association of malaria within similar age ranges: 5–14 years (OR 8.9 95% CI, 2.15–42.75), 15–29 years (OR 5,79 95% CI, 1.50–26.11), 30–44 years (OR 8.2 95% CI, 2.22–35.61), 45-64yrs (5.79 95% CI, 1.47–26.43). In their study, Tipmontree et al. [26], established a significant risk for malaria within the following age ranges 16–24 years (OR 2.28 95% CI,1.89–2.75), 25–40 years (OR 2.63 95% CI,2,27–3.16), 41–64 years (OR 1.73 95% CI 1.73 95% CI, 1.47–2.15) and 65+years (OR 0.68 95% CI, 0.46–0.98).

### Outdoor activity

Individuals who tested positive for malaria along the China-Myanmar border had a history of staying outdoors (OR 4.34 95%, CI 1.05–17.99) [26] as shown in Table 3. In Chipinge, along the Zimbabwe-Mozambique border, staying outdoors was associated with having malaria though significant (OR 1.56 95%, CI 0.6–3.5) [28]. Along the Namibia and Angola border, those who reported sleeping outside were more likely to contract malaria than those who did not (OR 5.61 95%, CI 1.97–16.02) [36]. Similarly, Kureya et al. in the Zimbabwe-Mozambique border region found an association between staying outdoors and having malaria, however, this association was not significant (OR 1.5 95%, CI 0.6–3.5) [28].

**Table 3:** Behavioural, occupation, and environmental risk factors.

Risk Factor	Significant Association	Negative Association	Non-significant Association
**Outdoor Activity**			
Outdoor Activity at night			1.5(0.6–3.5) [28]
Outdoor stay <7 days	4.34(1.05–17.99) [29]		
Outdoor stay >7days	4.13(1.29–13.13) [29]		
Having meals outside the house			0.6(0.3–1.5) [28]
Wearing protective clothes		0.5(0.26–95) [26]	
Wearing short clothes			0.8(0.3–1.8) [28]
**Use of Treated Nets**			
Use of net before malaria onset			0.76(0.52–1.12) [30]
Regular use of Insecticide Treated Nets (ITN)			1.69(0.92–3.12) [26]
Not having ITN at home	2.2(1.2–6.4) [33]		
Use of ITN		0.61(0.42–0.87) [27]	
ITN not hanged	2.7(1.2–6.4) [33]		
Non-use of bed net	5.49(4.12–7.32) [27]		
Sleeping under a net	0.65(0.35–1.75) [25]		
**Protective Measures**			
Use of measures against malaria	53.75(29.94–7.67) [30]		
Use of measures against mosquito		0.48(0.32–0.72) [30]	
Use of IRS household IRS in the past year		0.63(0.42–0.94) [27]	
Non-use of IRS	3.4(2.5–4.65) [27]		
**Proximity to Water bodies**			
Live within 100m of stream	17.62(7.17–45.27) [30]		
Live within 100m of a paddy field or pool	15.52 (4.27–66.72) [30]		
Living within 3km of a stream	4.7 (1.2–6.3) [28]		
**Housing Structure**			
Living in a house with open eaves	2.4 (1.0–5.6) [25]		
House made of pole and dagga			0.7(0.3–1.8) [28]
Sleeping in a house with open eaves	4.67 (2.14–10.57) [28]		
**Cross Border Movement**			
History of crossing the border into Mozambique	9.5(1.1–19.2) [28]		
Crossing from Guyana into Brazil	159.5 (75.1–338.9) [30]		
Crossing the border from Venezuela into Brazil	49.03(7.95–302.2) [33]		
Staying Overnight in Myanmar before illness	233.76(109.41-.43) [33]		
Recent mobility across the Myanmar border before the illness.	53.75(29.94–7.67) [30]		
Recent mobility across the Namibian border	5.95(1.68–21.08) [32]		

### Protective measures

Non-use of ITNs along the China-Myanmar border (OR 5.49 95%, CI 4.12–7.32) [26], not hanging an ITN (OR 2.7 95%, CI 1.2–6.4), and not having an ITN at home (OR 2.2 95% CI 1.2–6.4) [27] was associated with malaria infections. Within the Zimbabwe-Mozambique border zone, non-use of ITNs was significantly associated with malaria [28]. In the Namibia-Angola border region, the use of ITN was associated with less risk compared to non-use (OR 0.65 95%, CI 0.35–1.75) [32] though the association was non-significant. The use of preventative measures against malaria mosquitoes (OR 0.48 95% CI, 0.32–0.72) and IRS (OR 0.63 95% CI, 0.42–0.94) [31] were protective against malaria. In contrast, non-use of IRS was associated with the occurrence of malaria within the China-Myanmar border region (OR 3.42 95% CI 2.5–4.65). Similar observations were made in the Namibia- Angola border region [32].

### Occupation

Individuals who worked in forestry (OR 3.28 95%, CI 1.96–14.79), (OR 3.55 95%, CI 1.20 10.33) [27], (OR 2.66 95%, CI 1.95–3.62) [33] and mining (OR 55.76 95%, CI 50.59–61.46) [33] areas were at a greater risk of contracting malaria compared to those who did not. Smith et al. [32] in Namibia, reported significant risk for people engaged in night agricultural activities (OR 2.09 95%, CI 1.12–3.87) [32]. Being involved in tourism activities was also significantly associated with contracting malaria (OR 4.22 95%, CI 2.50–6.04) [33].

### Environment and living conditions

Individuals with residential places located closer to water bodies (OR 17.62 95%, CI 7.17–45.27) [30], paddies, and streams (OR 15.22 95%, CI 4.27–66.27) [30] were more likely to contract malaria compared to those living afar. Living within 3km of a stream was also associated with contracting malaria (OR 4.7 95%, CI 1.2–6.3) [28]. Those who owned houses with open eaves (OR 2.4 95% CI,1.0–5.6) [25] and those who reported sleeping in a house with open eaves (OR 4.67 95%CI, 2.4–10.57) [28] were significantly at risk of contracting malaria illness.

### Cross border movement

Across all the studies included in this review, individuals with a history of cross-border movement were at risk of contracting malaria. Within the Brazil-Venezuela border region, individuals from Venezuela (OR 233.76 95%, CI 109–199.43) [33] and Guyana (49.03 95%, 7.95–302.2) [33] were at risk of malaria. Along the China-Myanmar border regions, those with a history of staying overnight in Myanmar were at risk of contracting malaria (OR 53 95%, CI 29.94–97.67) [30]. Kureya et al. [28] also reported a risk of malaria among those with a history of traveling to Mozambique (OR 9.5 95%, CI 1.1–19.2). Moving across the border in Namibia was also significantly associated with the occurrence of malaria among travellers (OR 5.95 95% CI,1.68–21.08) [32].

### Meta-analysis

**Fig 2:**
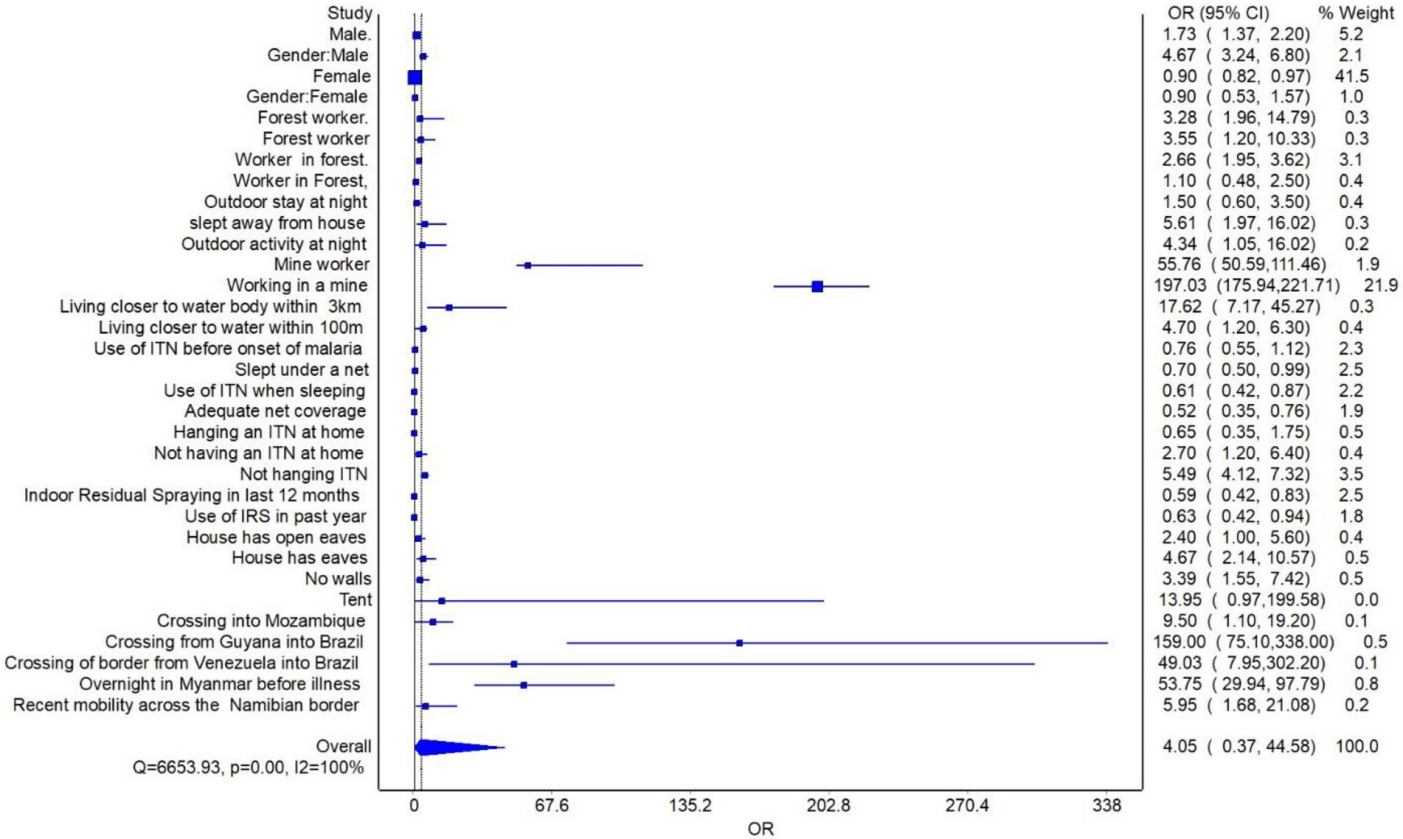
Forest plot for border malaria risk factors.

**Fig 3:**
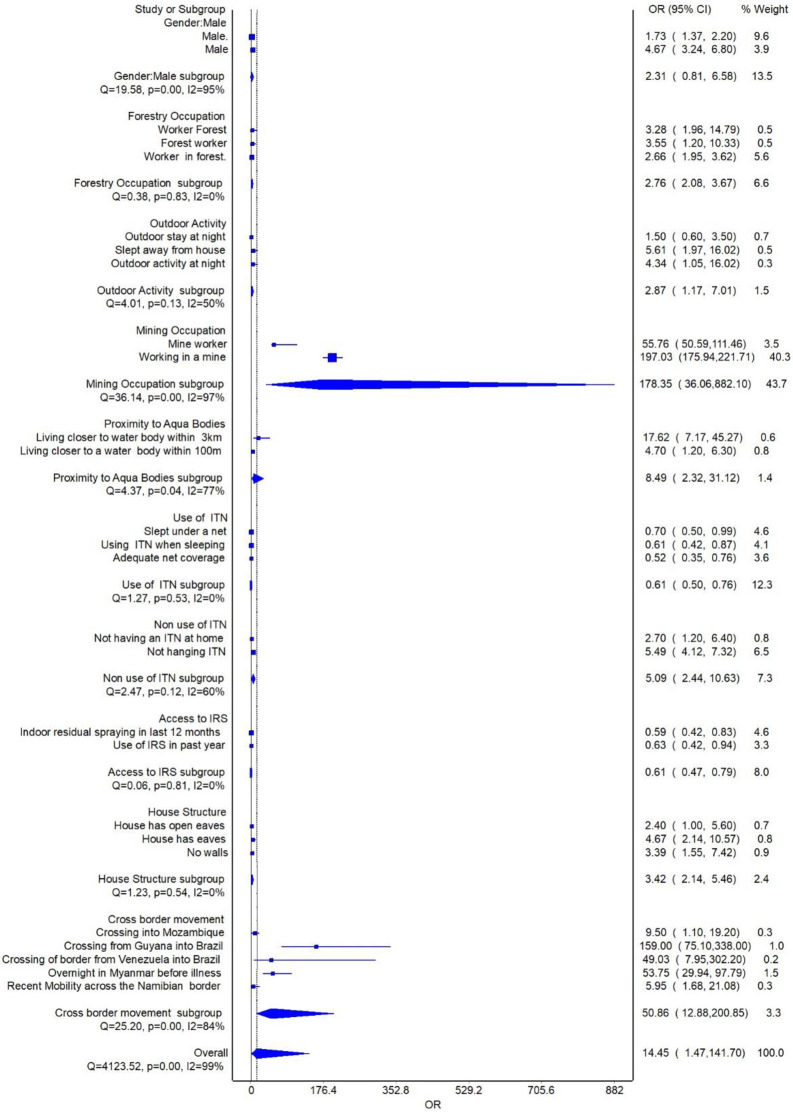
Forest plot for sub-group analysis.

Variables supported by two or more studies were meta-analyzed. Subgroup analysis was conducted by stratifying the main determinants into eleven groups. The pooled risk of contracting border malaria due to night outdoor movement increased the risk of contracting malaria by 2.87 times (95% C1: 1.17–7.01). Residing closer to an aquatic body increased the risk of malaria by 8,49 times (95% CI: 2.32–31.72) and non-use of ITNs (OR 5.09 95% CI, 2.44–10.63). The likelihood of contracting malaria within border regions due to poor housing structure increased by 3.57 times (95% CI: 2.25–5.56). Moving across the border increased the risk of contracting malaria by 50.86 times (95% CI: 2.88–200.85) Fig 2. Increased risk of contracting malaria was also associated with working in forestry (OR 2.76 95% CI,2.08–3.67) and mining (197 95% CI, 175–221.71) within border regions. Access and utilisation of ITNs reduced the risk of malaria by 35% (OR 0.65 95% CI, 0.54–0.77). Additionally, living in spaces subjected to IRS was protective (0.61 95%CI, 0.47–0.79). A sensitivity analysis was also conducted to identify sources of heterogeneity. The model was reduced by excluding non-significant factors. The risk of border malaria due to outdoor night activity, cross-border movement, living closer to aquatic bodies, non-use of ITN, and poor housing structure remained relatively unchanged Fig 3. The overall risk for border malaria increased from 4.05 times to 14.45 times. Heterogeneity in this review remained high despite further sensitivity analysis (I^2^ >99%). The high heterogeneity observed can be attributed to differences in settings, methodologies, study designs, and study participants. The LFK index on the Doi plot showed no asymmetry denoting a lack of publication bias S1 Fig.

## Discussion

We conducted this global systematic and meta-analysis to establish the aggregated risk of contracting malaria within border regions. Border malaria remains a significant impediment to the attainment of malaria elimination. This study identified night outdoor activity, working in forestry and mining activities, poor housing, living closer to water bodies, and cross-border movement as key drivers for border malaria. The use of ITNs and IRS was protective. This study reveals a comparability of border and non-border malaria risk factors. Our results suggest a need to sustain integrated malaria control within border regions and cross-border control alliances to mitigate against border malaria.

Our study revealed a contrast in risk for malaria for both males and females in different regions. Several studies have also shown this differential risk for males and females [39–41]. Social roles, level of education, occupations, access to health information, incomes, and knowledge levels could be possible explanations for this risk variation among males and females [40, 42–44]. Occupation and social roles for both males and females likely reduce or elevate vector-human contact. Malaria interventions should therefore be alert to gender, occupation, and social roles of key population groups. Diiro et al. [42] argue for the universal promotion of inclusive access to health information, equal opportunities, and access to integrated malaria prevention interventions to reduce malaria risk in endemic settings.

Our review showed the risk for malaria for selected age groups within the 0–64 years age range. The magnitude of malaria risk in specific age strata varied across studies. This variation in risk could be explained by differences in socioeconomic status, occupation, individual behaviour, access to health information, and quality of housing attributes as shown in other studies [45–47]. Thus, interventions seeking to address age as a risk factor should be targeted at the most at-risk group within specific geographical regions to optimize resources and coverage.

Night outdoor activity was associated with the occurrence of malaria in this review. Individual outdoor activity for social and economic reasons that coincide with peak biting periods increases the likelihood of human vector contact. Mosquito biting behaviour and biting rates have been shown to peak during the early evening and early morning hours when human activity is also likely to be high in some settings [48–50]. In other geographical settings, biting rates have also been shown to be evenly distributed throughout the whole night [51] thereby posing a constant risk to individuals who engage in outdoor activity at night. Wearing long clothes and sleeping in rooms subjected to IRS has been shown in this study and elsewhere to be an effective protective intervention against mosquito bites [52, 53]. Risk communication messages should be targeted at limiting night outdoor activity with an emphasis on staying indoors in sprayed spaces. Protection during night door activity is available through wearing long clothes and this low-cost intervention should be amplified.

This study reinforces the critical protective role of IRS and ITNs. IRS and ITNs form the core vector control interventions that have been responsible for the reduction in global malaria burden [54, 55]. Non-use of ITNs as revealed in this study is a public health concern. Non-use of ITN has been linked to limited knowledge, low coverage, and limited access to adequate nets per household population [56–58]. It is critical for border regions’ malaria programmatic planning to integrate strong advocacy and community engagement activities which will increase treated net utilisation. Ensuring universal coverage of ITNs through routine health services and integrated community health outreach programs could be pivotal.

The presence of water bodies in addition to altitude and temperature is essential for sustaining the mosquito life cycle [59]. Residing closer to water bodies likely increases the likelihood of human and vector interaction. Evidence has shown increased risk posed to individuals who reside closer to water bodies such as rivers, streams, and paddies [60] as studies have shown mosquito mobility capabilities of up to 3km [61–64]. Environmental management of breeding sites in addition to IRS and ITNs within border regions utilising larval source management techniques [65] should be promoted to interrupt mosquito lifecycle and reduce mosquito density [66].

As shown in this study, housing structure and quality play a critical role in malaria transmission within border communities. Houses made of poles and dagga and with open eaves; were significantly associated with contracting malaria similar to findings by Mundagowa and Chimberengwa [67]. Eaves allow mosquito entrance into sleeping spaces, thereby increasing the risk of mosquito bites and reducing the protective effectiveness of IRS. Individuals with access to modern housing have been shown to have a lower risk of contracting malaria compared to those living in poorly structured traditional houses [68]. Improving access to quality housing and closing eaves, screening doors and windows can limit vector entrance into residential spaces [69]. Poor housing structures could indicate low economic status for communities living in border regions. Improving the socio-economic status of communities within border regions could also be pivotal in reducing border malaria risk [70].

Our study revealed elevated risk for those engaged in forestry, agriculture, and mining. Forestry workers stay longer in outdoor environments and are often accommodated in makeshift and poor housing structures, increasing their vulnerability to mosquito bites [52]. Additionally, farming activities are often associated with irrigation systems, canals, and activities that promote the formation of water puddles and small ponds that act as breeding sites [60]. Water-logged fields provide breeding spaces for vectors and these are often associated with higher larval and adult mosquito densities [48]. Studies conducted along the Thai-Myanmar border region [71] and the Brazil-Venezuela-Guyana tri-border region [34] revealed an elevated risk for forestry and agricultural workers. Measures that protect forestry, agricultural, and mining workers along borderlines should be developed. A study by Ngninpognieta et al. [72] in Cameroon, revealed poor utilization of ITNs among similar workforce. Sannan et al. [52] in their study showed that workers engaging in agricultural activities were willing to take up protective measures against malaria. Provision of ITNs, health information, encouraging workers to wear long clothes during the night, and provision of better housing units may likely reduce the risk of contracting malaria among communities working within the forestry and agricultural sectors.

Human travel poses complex challenges to malaria control with its ability to move parasites across borders and sustain transmission in low transmission zones nearing elimination [34, 73] [74]. The movement of individuals from non-endemic regions coupled with limited access to preventative methods and health information could likely explain the elevated risk [75]. Population movement from non-endemic regions to endemic regions coupled with exposure to highly efficient vectors has been shown to sustain malaria transmission along border regions [59]. THPM is a permanent feature largely driven by social, economic, and political push factors, hence there is a need to develop strategies that promote access to malaria-preventative methods for mobile populations [76]. Increasing access to preventative interventions such as chemoprophylactic drugs, health education, and provision of ITNs and repellents [75, 77] may mitigate against risk of malaria as this has the potential to reduce parasite movement and importation [78–81]. Implementing a voluntary border screening program at points of entry has been recommended elsewhere [77], however, this initiative may be threatened by travellers’ non-compliance and limited human resources for health at points of entry (PoE) as revealed in a study by Munsense et al. [19]. The existence of illegal and informal PoEs in many regions globally further threatens the effectiveness of such initiatives. These multiple threats call for enhancing regional and multi-sectoral alliances that address cross-border malaria [82].

Risk factors such as night outdoor activities, occupation in agriculture, and forestry activities, and the protective effect of wearing long clothes and IRS and ITN have been established in the literature. Similarities between border and non-border malaria risk factors are evident in this review. The similarities have positive implications for malaria control activities as established interventions will be applicable. Similar to the observation by Li et al. [70], we argue that interventions that have proved effective for non-border malaria are effective in border regions. Whilst the guiding document from WHO defines border malaria and cross-border malaria; it does not offer clarity on “specific distance from border line” [5]. The lack of defining distance parameters in the current global border malaria working definition may lead to inconsistency and misclassification. We recommend a further review of the working definition of border malaria to allow a clearer distinction between border and non-border malaria.

This study had limitations which need to be considered. This review only considered articles published in English peer reviewed journals. The excluded studies could likely have reduced or increased the effect size estimates obtained in this analysis. The varied reporting of age strata could not allow for the pooling of age in the meta-analysis. The high level of heterogeneity as revealed in this review has implications for the generalisation of the results. Therefore, findings from this review should not be taken as definitive drivers but as indicative drivers of border malaria. The likelihood of misclassification of border malaria is high as the papers reviewed did not report on the association of malaria and distance from the border region. Eligible papers included in this review did not report on vectoral bionomics within the border regions. Understanding vectoral behaviour is critical in developing integrated malaria control interventions suitable for border regions. Despite these limitations, this paper possesses some strength in that it includes studies from various malaria-endemic border regions of the world with ongoing transmission. Therefore, it offers a global perspective of the drivers of malaria within border regions and the findings are useful in generating context-specific interventions.

## Conclusion

This review confirms the significance of outdoor night activity, outdoor occupations, poor housing, proximity to aquatic bodies, and movement across borders as drivers for border malaria. Malaria control interventions such as IRS and ITNs use, remain effective and viable protectors against malaria within border regions. The complexities associated with border regions present notable challenges which require integrated and targeted approaches that address multifaceted social, behavioural, economic, and environmental determinants. Improving access to quality housing, behaviour change messages, and personal protective measures in risky occupations can reduce border malaria. The heterogeneity of risk evident in this review calls for local and regional interventions appropriate to address local malaria transmission dynamics and population behaviour. It is evident from this review that border malaria risk factors are comparable to non-border malaria drivers. Interventions such as IRS and ITNs that have proved effective in control programs for non-border malaria must be sustained whilst formulating novel interventions that address peculiar risks like cross-border human population movement. Therefore, interventions targeted at mobile populations should be instituted through multi-country and multi-sectoral cross-border malaria control alliances.

## Supporting information

S1 Table Articles Included in the systematic and meta-analysis. (DOCX)

S2 Table Results from literature search. (XLSX)

S3 Table Exposure variables associated with border malaria. (XLSX)

S1 Fig Doi plot showing level of asymmetry. (TIF)
