# Supplementary material for: Factors associated with contracting border malaria: A systematic and meta-analysis
Source: PLoS One. 2025 Jan 3;20(1):e0310063. doi: 10.1371/journal.pone.0310063 (PMC11698403; doi:10.1371/journal.pone.0310063)
Supplement: S1 Table — (DOCX) [file pone.0310063.s001.docx]

| **No** | **Author and year** | **Q1** | **Q2** | **Q3** | **Q4** | **Q5** | **Q6** | **Q7** | **Q8** | **Quality Index** |
| --- | --- | --- | --- | --- | --- | --- | --- | --- | --- | --- |
| 1 | Kanyangarara et al. (2016) (25) | 1 | 1 | 1 | 1 | 1 | 1 | 1 | 1 | 1 |
| 2 | Tipmore et al. (2009)(26) | 1 | 1 | 1 | 1 | 0 | 0 | 1 | 1 | 0.75 |
| 3 | Zhao et al. (2018)(27) | 1 | 1 | 1 | 1 | 1 | 1 | 1 | 1 | 1 |
| 4 | Kureya et al. (2017)(28) | 1 | 1 | 1 | 1 | 1 | 0 | 1 | 1 | 0.88 |
| 5 | Chavepojnkamjon et al. (2004)(29) | 1 | 1 | 1 | 1 | 1 | 1 | 1 | 1 | 1 |
| 6 | Xu et al. (2015)(30) | 1 | 1 | 1 | 1 | 0 | 0 | 1 | 1 | 0.75 |
| 7 | Li et al. (2013)(31) | 1 | 1 | 1 | 1 | 1 | 1 | 1 | 1 | 1 |
| 8 | Smith et al. (2021)(32) | 1 | 1 | 1 | 1 | 1 | 1 | 1 | 1 | 1 |
| 9 | Arisco et al. (2021)(33) | 1 | 1 | 1 | 1 | 1 | 1 | 1 | 1 | 1 |
| 10 | Wangdi et al (2022) (34) | 1 | 1 | 1 | 1 | 1 | 1 | 1 | 1 | 1 |
| 11 | Tejedor-Garavito et al. (2017)(35) | 1 | 1 | 1 | 1 | 1 | 1 | 1 | 1 | 1 |
| 12 | Smith et al. (2017)(36) | 1 | 1 | 1 | 1 | 1 | 1 | 1 | 1 | 1 |

**Assessment Questions**

|  | Yes | No | Unclear |
| --- | --- | --- | --- |
| 1. Were the criteria for inclusion in the sample clearly defined? | □ | □ | □ |
| 1. Were the study subjects and the setting described in detail? | □ | □ | □ |
| 1. Was the exposure measured in a valid and reliable way? | □ | □ | □ |
| 1. Were objective, standard criteria used for measurement of the condition? | □ | □ | □ |
| 1. Were confounding factors identified? | □ | □ | □ |
| 1. Were strategies to deal with confounding factors stated? | □ | □ | □ |
| 1. Were the outcomes measured in a valid and reliable way? | □ | □ | □ |
| 1. Was appropriate statistical analysis used? | □ | □ | □ |
