# Supplementary figures and images for: Factors associated with contracting border malaria: A systematic and meta-analysis
Source: PLoS One. 2025 Jan 3;20(1):e0310063. doi: 10.1371/journal.pone.0310063 (PMC11698403; doi:10.1371/journal.pone.0310063)

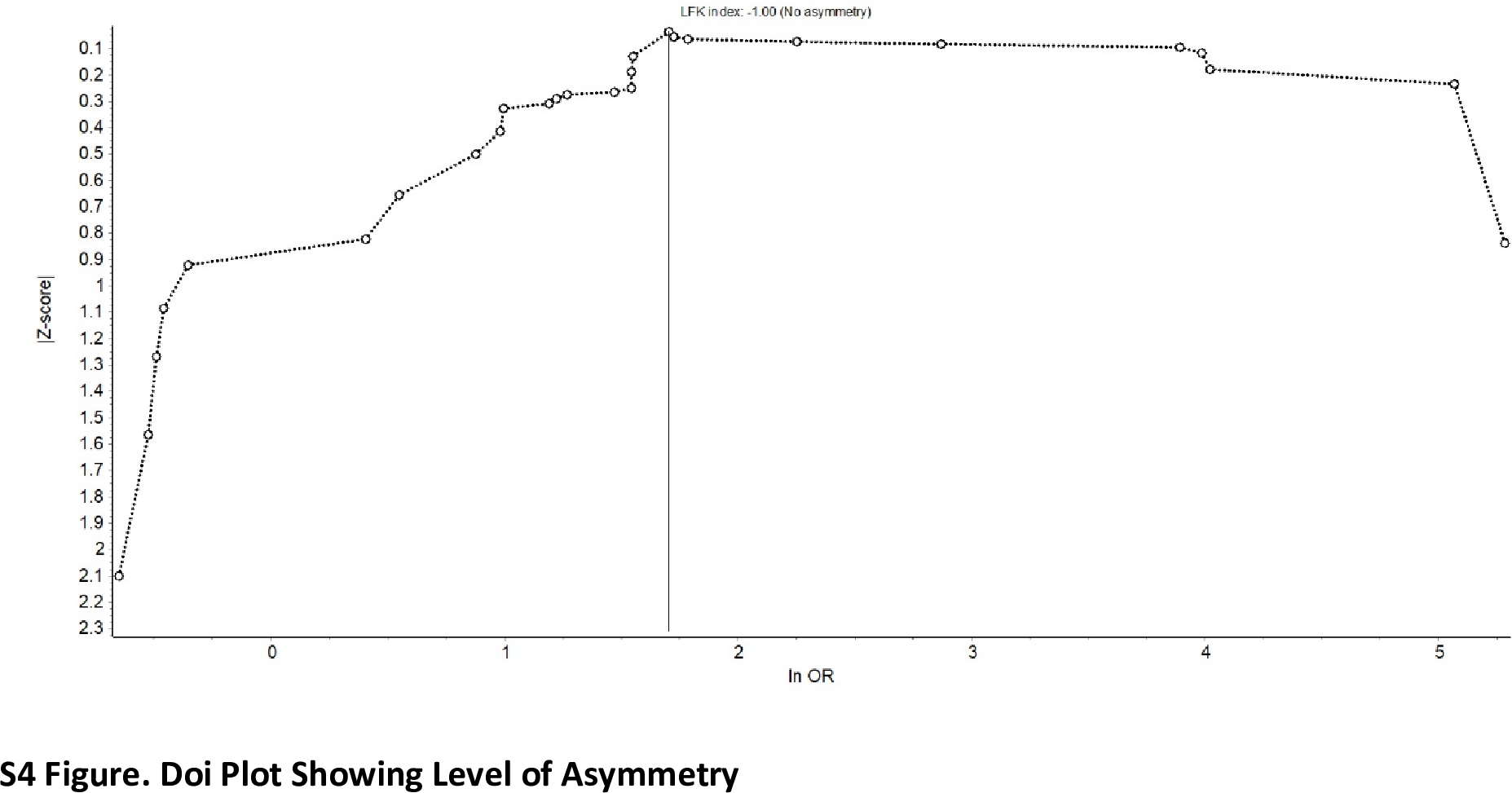

Supplement: S1 Fig — (TIF) [file pone.0310063.s004.tif]
